# Exogenous zinc mitigates salinity stress by stimulating proline metabolism in proso millet (*Panicum miliaceum* L.)

**DOI:** 10.3389/fpls.2023.1053869

**Published:** 2023-03-10

**Authors:** Naveed Ul Mushtaq, Khalid M. Alghamdi, Seerat Saleem, Inayatullah Tahir, Ahmad Bahieldin, Bernard Henrissat, Mohammed Khalid Alghamdi, Reiaz Ul Rehman, Khalid Rehman Hakeem

**Affiliations:** ^1^ Department of Bioresources, School of Biological Sciences, University of Kashmir, Srinagar, India; ^2^ Department of Biological Sciences, Faculty of Science, King Abdulaziz University, Jeddah, Saudi Arabia; ^3^ Department of Botany, School of Biological Sciences, University of Kashmir, Srinagar, India; ^4^ UMR7257 CNRS - Aix-Marseille University, Marseille, France; ^5^ Princess Dr. Najla Bint Saud Al-Saud Center for Excellence Research in Biotechnology, King Abdulaziz University, Jeddah, Saudi Arabia; ^6^ Department of Public Health, Daffodil International University, Dhaka, Bangladesh

**Keywords:** P5CS, zinc, proline, salt stress, millets

## Abstract

Salinity is one of the most concerning ecological restrictions influencing plant growth, which poses a devastating threat to global agriculture. Surplus quantities of ROS generated under stress conditions have negative effects on plants’ growth and survival by damaging cellular components, including nucleic acids, lipids, proteins and carbohydrates. However, low levels of ROS are also necessary because of their role as signalling molecules in various development-related pathways. Plants possess sophisticated antioxidant systems for scavenging as well as regulating ROS levels to protect cells from damage. Proline is one such crucial non-enzymatic osmolyte of antioxidant machinery that functions in the reduction of stress. There has been extensive research on improving the tolerance, effectiveness, and protection of plants against stress, and to date, various substances have been used to mitigate the adverse effects of salt. In the present study Zinc (Zn) was applied to elucidate its effect on proline metabolism and stress-responsive mechanisms in proso millet. The results of our study indicate the negative impact on growth and development with increasing treatments of NaCl. However, the low doses of exogenous Zn proved beneficial in mitigating the effects of NaCl by improving morphological and biochemical features. In salt-treated plants, the low doses of Zn (1 mg/L, 2 mg/L) rescued the negative impact of salt (150mM) as evidenced by increase in shoot length (SL) by 7.26% and 25.5%, root length (RL) by 21.84% and 39.07% and membrane stability index (MSI) by 132.57% and 151.58% respectively.The proline content improved at all concentrations with maximum increase of 66.65% at 2 mg/L Zn. Similarly, the low doses of Zn also rescued the salt induced stress at 200mM NaCl. The enzymes related to proline biosynthesis were also improved at lower doses of Zn. In salt treated plants (150mM), Zn (1 mg/L, 2 mg/L) increased the activity of P5CS by 19.344% and 21%. The P5CR and OAT activities were also improved with maximum increase of 21.66% and 21.84% at 2 mg/L Zn respectively. Similarly, the low doses of Zn also increased the activities of P5CS, P5CR and OAT at 200mM NaCl. Whereas P5CDH enzyme activity showed a decrease of 82.5% at 2mg/L Zn+150mM NaCl and 56.7% at 2mg/L Zn+200 mM NaCl. These results strongly imply the modulatory role of Zn in maintaining of proline pool during NaCl stress.

## Introduction

Plants are impacted by both biotic and abiotic stress conditions which inhibit the uptake of water and nutrients, compromise membrane permeability and hamper development (Arif et al., 2020). These alterations also affect the metabolism of hormones, and the exchange of gasses and result in the production of ROS at a faster rate. The salinity stress reduces primary photochemistry of photosystem and reduced photosynthetic pigments, exhibited enhanced chorophylll degradation and leakage of electrolyts (Siddiqui et al., 2019; Akhter et al., 2021). Continuous exposure to such conditions finally causes plant senescence and death (EL Sabagh et al., 2021). Salt stress in plants is one of the most significant ecological restrictions influencing plant growth and development which poses a devastating threat to global agriculture (Mushtaq et al., 2021). Worldwide, the rate of salinity is high which affects approximately 20% of the world’s land and it has been steadily increasing for a few decades (Khan et al., 2022). The overuse of fertilizers and outdated irrigation practices are primarily to blame for excessive salt levels in agricultural lands (Ladeiro, 2012). An excessive amount of salt causes hyperosmotic and hyperionic conditions, accumulation of Na^+^ and Cl^-^ ions and the generation of ROS (Rahman et al., 2016). The increased ROS quantities impact the plants negatively by damaging cellular components, including nucleic acids, lipids, proteins, and carbohydrates (Das and Roychoudhury, 2014; Saleem et al., 2021). However, moderate levels of ROS are necessary as they function as a signalling molecule (Mittler, 2017; Marcec and Tanaka, 2021). On the other hand, plants have a sophisticated antioxidant system that scavenges and regulates the levels of ROS to protect cells from damage (Kapoor et al., 2019). Proline is one such crucial non-enzymatic osmolyte that plays a function in stress reduction (Alamri et al., 2019; Iqbal et al., 2021). Besides its role in plant improvement, proline is also involved in flowering, pollen, embryos, and leaf growth. As a response to stress, proline is typically boosted in the cytosol to regulate the osmotic environment (Meena et al., 2019). Apart from its function as an osmolyte, it also works as a metal chelator and antioxidant molecule during stressful situations (Hayat et al., 2012). Proline accumulation enhances heavy metal tolerance, and improved resistance to drought or salinity stress in plants and algae (Hmida-Sayari et al., 2005; Zhao et al., 2022). There has been extensive research on improving the tolerance, effectiveness, and protection of plants against stress, and to date, various substances have been used to mitigate the adverse effects of salt. Microelements are thought to help plants cope with salt stress (Abideen et al., 2022) and throughout their life cycle, plants require these elements to survive in contrasting environmental conditions. Deficiencies of these elements can significantly affect a plant’s growth, development, and survival. In the context of requirement, certain elements may not be required by all the plants, but are advantageous to particular plant species, and are therefore called beneficial elements. Beneficial elements consist of zinc (Zn), cobalt (Co), selenium (Se) and silicon (Si) (Kaur et al., 2016). Research indicates that these elements are beneficial to plant growth and development in both optimal and stressful environments. In order to enable plants to cope with stress adversities and survive, beneficial elements regulate essential acclimation responses through molecular, physiological, and biochemical mechanisms (Kumari et al., 2022). They increase abiotic stress tolerance in plants by an intricate crosstalk with other plant growth regulators such as phytohormones, ROS and other signalling molecules (Tiwari et al., 2017; Khan et al., 2021). However, their beneficiary and essentiality is debatable, with little evidence indicating necessity. In the era of climate change, a restored understanding of beneficial elements may also be beneficial to improving stress tolerance, plant health, plant nutritional value and crop productivity. As a result, the principles behind the impacts of beneficial components in plants need to be explored, and the field provides a chance to gain more insights that might aid in achieving sustainable agricultural yield and plant adaptation to abiotic stress conditions. Micronutrients such as boron (B), chloride (Cl), copper (Cu), iron (Fe), manganese (Mn), molybdenum (Mo), nickel (Ni), and zinc (Zn) are required in much lesser amounts by the plant (Thapa et al., 2021). It is difficult to specify the precise numbers of micronutrients since certain elements are still not clearly classified as essential or beneficial. 17 of the 92 natural elements found in plants are considered essential nutrients. Among these 17 elements, 8 are micronutrients which include iron (Fe), zinc (Zn), copper (Cu), manganese (Mn), molybdenum (Mo), chlorine (Cl), boron (B), and nickel (Ni) (Mondal and Bose, 2019). Micronutrients are involved in almost all metabolic and cellular activities, including primary and secondary metabolism, energy metabolism, cell defense, gene regulation, hormone sensing, signal transduction, and reproduction. Micro nutrients also play vital roles in plant growth, development and food grain production, enhancing tolerance to abiotic stress, maintaining water potential, provide protection against environmental extremities and pathogen (Tripathi et al., 2015; Chrysargyris et al., 2022).

Zinc is one of the cardinal micronutrients which mitigates stress in plants (Dimkpa et al., 2019; Venugopalan et al., 2022). Studies reported that the use of Zn has improved plant growth, pigment content, carbohydrates, proteins, antioxidants, and the plant defence system (Noreen et al., 2021; Shah et al., 2022; Wei et al., 2022). It aids in membrane stability, hormone production, starch and sucrose turnover, RNA and DNA structure stabilization, gene expression, auxin formation, photosynthesis, and protection against drought, cold, salt, and pathogens (Umair Hassan et al., 2020; Hassanein et al., 2021; Rai-Kalal and Jajoo, 2021). Zinc deficiency in rice was reported to mediate the induction of CAZymes (Carbohydrate-Active enZymes) involved in starch synthesis/transport *via* up-regulation of genes encoding these CAZymes (Suzuki et al., 2012). These enzymes mainly belong to CAZy classes glycoside hydrolase (GH) and glycosyltransferases (GT) (Diricks et al., 2015; Stam et al., 2006) (http://www.cazy.org). Interestingly, the ability of rice plants to withstand the low level of Zn in their cells is prompted by the accumulation of starch mediated by certain CAZymes of the Kyoto Encyclopedia of Genes and Genomes (KEGG) pathway “Sucrose and Starch Metabolism” (map00500). These CAZymes include 4-alpha-glucanotransferase (EC 2.4.1.25) and 1,4-alpha-glucan branching enzyme (EC 2.4.1.18) that act on the transfer of α-1,4-glucosidic bond, respectively, from maltose to amylose, and eventually to starch (https://www.brenda-enzymes.org/) (Lombard et al., 2014).

The normal concentration of Zn in most plants is between 25 to 150 ppm, however, this small amount of Zn plays a key role in more than 300 enzymes, such as alkaline phosphatase, carbonic anhydrase, alcohol dehydrogenase, and Cu-Zn superoxide dismutase (Malik et al., 2011; Solanki, 2021). It also has a structural role in the stabilization of proteins such as Zn cluster, Zn finger and RING finger domains/motifs. In crop plants, zinc is transported directly from the soil either in Zn^2+^ form and get accumulated in the roots of plants before being translocated to the shoots and leaves *via* xylem (Vatansever et al., 2017). In plants, Zn homeostasis is maintained by ZIP (Zn, iron-permease family/ZRT, IRT proteins) family uptake transporters in a coordinated regulation mechanism. Other proteins involved in the translocation of Zn are the heavy metal ATPase (HMA) family and the metal tolerant proteins (MTP) family (Olsen and Palmgren, 2014). ZIP family participates in the Zn influx into cell cytosol, while HMA mediates Zn efflux into the apoplast. Zn sequestration into the vacuoles and endoplasmic reticulum are facilitated by the MTP family (Gupta et al., 2016).

We hypothesized that a stressor (NaCl) and a mitigant (Zn) would have an impact on the proline biosynthesis which would reflect as a response on plant fitness. Thus to understand this process

we aimed to visualize the merit of using Zn as a mitigant against salt stress (NaCl) and to understand the role of Zn in regulating proline pathway under salt stress in proso millet. Thus we studied proline metabolism, proline accumulation and enzyme activities related to proline biosynthesis. We also elucidated plant growth parametrs after the application of zinc (zinc sulfate).

## Material and methods

### Plant growth and treatments

The seeds of proso millet (*Panicum miliaceum* L.) were collected and identified at the Centre for Biodiversity and Taxonomy, University of Kashmir. The seeds were sterilized using 70% (v/v) ethanol for 1 minute and washed with sterile distilled water. Surface sterilization of seeds was performed using 10% sodium hypochlorite solution for 10 min followed by rinsing with sterilized distilled water. The seeds were sown in pots, 20 cm in diameter and containing autoclaved sand. Each pot of specific diameter contained 900grams of sand and 1gram of seeds (approx. 500 seeds). A controlled environment with a 26 ± 1°C temperature and a 16-h photoperiod was maintained (Khalid et al., 2008; Desoky et al., 2019). Three sets of plants were grown with three replicates each and the treatments viz., 0, 150 and 200 mM of NaCl were given to the pots as per (Shah et al., 2020) and the Zinc (Zn) treatments 1 to 5 mg/L were provided in the form of zinc sulfate (ZnSO_4_). The Hoagland’s nutrient medium (pH 6.5) containing all macro and micro nutrients was used as a nutrient source for the growth of plants. Till 14^th^ day of sowing, plants were nourished with Hoagland’s nutrient medium and afterwards both salt and Zinc were applied with nutrient solution. For maintaining concentrations of treatments throughout the stress period, treatments were repeated every third day till harvesting. The experiment was performed in a complete randomized design and each treatment was replicated three times (**Supplementary Table 1**). The plants were harvested after 22 days of sowing for morphological (Shoot length, root length, total length, leaf height and area), physiological and biochemical analysis.

### Determination of plant growth parameters and tolerance index

Following the experiment, 10 plants were taken at random from each treatment and gently cleansed four times with deionized water to remove adherent sand from the root surfaces. Following this, the morphological parameters were examined (Singh et al., 2008; Kausar et al., 2012; Dikobe et al., 2021). To evaluate the capacity of plants to thrive under high saline environments, the tolerance index (TI) was calculated by the equation given by Wilkin (Wilkins, 1957):


Tolerance Index(TI %)=MLT/MLC×100

MLT = Mean length (root, shoot) of the longest root/shoot in treated plants, MLC = Mean length (root, shoot) of longest root/shoot in control.

### Fresh weight/dry weight/relative water content

The biomass accumulation (BA) of 10 plants was determined by drying in an incubator at 70°C for 48 hours. To calculate relative water content (RWC), the fresh weight (FW) of leaves was taken. Following this, the dry weights (DW) of leaves were taken by drying in an oven at 70°C for 48 h (Afzal and Mansoor, 2012). RWC was analyzed using the formula:


RWC (%) =FW−DW/FW×100

### The membrane stability index and electrolyte leakage

The membrane stability index (MSI) and Electrolyte leakage (EL) was determined by the method of Singh (Singh et al., 2008). Leaves were sterilized 3 times with distilled water before being chopped into small pieces and put in vials containing 10 mL of double distilled water. For the initial electrical conductivity of the solution (EC1), the vials were placed in a water bath at 40°C for 30 minutes. To obtain the final electrical conductivity (EC2), the vials were subjected to boiling temperature in a water bath for 10 minutes and then allowed to cool before taking EC2 readings. The EL and MSI were measured by using the following formulae;


EL (%)=(EC1/EC2)×100

MSI (%)=[1− (EC1/EC2)]×100

### Photosynthetic pigments and chlorophyll stability index

0.2g of leaf sample was homogenized in 10ml of 80% acetone under dark conditions. Total chlorophyll, chlorophyll a and chlorophyll b were measured following the standard methods (Lichtenthaler, 1987; Hou et al., 2018);. For calculating anthocyanin, the pre-frozen leaf samples (0.1g) were homogenized in 10 ml of acidified methanol (methanol, double distilled water and concentrated HCl in the ratio of 80:20:1) in dark conditions (Benazzouk et al., 2020). Carotenoids, total phenolics and total flavonoid content were calculated as per Golkar and Taghizadeh and Benazzouk et al. (Golkar and Taghizadeh, 2018; Benazzouk et al., 2020). The stability of chlorophyll was measured by the chlorophyll stability index (CSI) as per Sairam et al. (Sairam et al., 1997) by the following formula;


CSI=(Total Chl. under stress/Total Chl. under control)×100

### 2, 2-Diphenyl-1-Picrylhydrazyl activity

The DPPH-radical scavenging activity was calculated using the method given by Sethi et al. (Sethi et al., 2020). To measure the radical scavenging activity of the methanolic extract, 0.1 ml of extract was allowed to inhibit 3.9 ml of DPPH. UV-VIS spectrophotometer was used to measure the absorbance of the reaction mixture at 517 nm and the percentage of DPPH radical scavenging activity was calculated by the following equation:

The percentage inhibition (IP) of absorbance was determined using the following equation:


$$IP\;\left(\%\right)=\left[A_{control}-\;A_{sample}/A_{control}\right]\;\times \;100$$

Where, A_control_ is the absorbance of the control reaction and A_sample_ is the absorbance in the presence of a methanolic sample.

### Ferric reducing antioxidant power

The antioxidant capacity of the samples was determined spectrophotometrically using the method of Rajurkar and Hande with some modifications (Rajurkar and Hande, 2011). At low pH the electron donating antioxidants reduction of Fe^3+^ TPTZ complex (colourless complex) to Fe^2+^ -tripyridyltriazine (blue coloured complex) takes place which was read at 593 nm after 4 minutes. The sample (10 µl) was added to a 300 µl FRAP reaction mixture containing 300 mM acetate buffer, 10 ml TPTZ in 40 mM HCl and 20 mM FeCl_3_ in the proportion of 10:1:1 at 37°C. Ferrous ammonium sulphate was used as a standard for calculating FRAP activity.

### Proline content estimation

The ninhydrin method was used to assess the proline content of the leaves as per Zhu et al. (Zhu et al., 2020) with some modifications. Leaves (0.5 g) collected were extracted in 3 percent (w/v) sulfosalicylic acid. The leaves were weighed and finely grounded using liquid nitrogen. The mixture was kept as such for a few minutes and was centrifuged at 12,000 g for 10 min. The supernatant obtained after centrifuge was used to estimate proline content. The supernatant was combined with 2 ml of acid ninhydrin and 2 ml of glacial acetic acid and placed in a 100°C water bath for 1 hour. The reaction was stopped by immersing the test tubes in an ice bath. Further 4 ml toluene was added to the mixture and the absorbance at 520 nm was measured with a spectrophotometer. The content of proline was measured using a proline standard curve made with different concentrations.

### Enzyme extraction and assays

To find out the activity of proline metabolism enzymes, the leaf samples was homogenized in an extraction buffer containing 100 mM Tris-HCl, 1 mM EDTA, 10 mM MgCl2, 10 mM β-mercaptoethanol, 2 mM PMSF, 4 mM DTT, and 2% PVPP (pH 7.5). The homogenate was centrifuged at 4^°^C at 10,000 g for 20 min. The supernatant was stored at -80^°^C for enzyme assays. Pyrroline-5-carboxylate synthase (P5CS), Δ-pyrroline-5-carboxylate reductase (P5CR), δ-ornithine amino transferase (OAT), Δ-pyrroline-5-carboxylate dehydrogenase (P5CDH) and proline dehydrogenase (ProDH) assays were performed following standard protocols with some modifications (Parida et al., 2008; Spoljarevic et al., 2011; Da Rocha et al., 2012; Koenigshofer and Loeppert, 2019; Zhu et al., 2020)

### Pyrroline-5-carboxylate synthase activity

This study determined the P5CS activity based on the utilization of NADPH during the reaction catalyzed by the enzyme. At 25°C, the P5CS activity was performed in a final volume of 2 mL of 100mM Tris-HCl buffer (pH 7.5) containing 25mM MgCl_2_, 75mM Na-glutamate, 10mM ATP, 0.4mM NADPH, and the enzyme extract. Using UV–Vis spectrometer, NADPH consumption was monitored as a decrease in absorption at 340 nm as a function of time.

### Δ-pyrroline-5-carboxylate reductase activity

P5CR activity was determined by measuring the proline-dependent reduction of NAD^+^ (the reverse reaction). At 25°C, the reaction was performed in a final volume of 2 mL of 200mM sodium glycinate buffer (pH 10.3), 20mM proline, 15mM NAD^+^ (pH 5-7), and the enzyme extract. To measure the formation of NADH, absorbance at 340 nm was monitored by using UV-Vis spectrometer.

### Ornithine amino transferase activity

To determine the activity of δ-OAT, pyrroline 5-carbuxylate (P5C) was measured for 30 minutes using the ninhydrin method. In a final volume of 1 mL, the reaction mixture contained 100 mM Tris-HCl (pH 8.0), 20 mM α-ketoglutarate, 50 mM L-ornithine, and the enzyme extract. The mixture was incubated for 30 min at 37°C. Using 0.2 mL of 2% (w/v) ninhydrin and 3 N perchloric acids the reaction was stopped. After incubating at 100°C for 5 min and centrifugation at 12,000*g* for 10 min, the precipitation was dissolved in 1.5 mL of ethanol. Now, the mixture was incubated at 100°C for 5 minutes. Following this, 10 minutes of centrifugation at 12,000g was carried out and the precipitate obtained was dissolved in 1.5 mL of ethanol. The clear supernatant was read at 510nm using UV-VIS spectrophotometer. One unit of δ-OAT activity was represented as the micromoles of P5C formed per mg of protein per hour.

### Δ-pyrroline-5-carboxylate dehydrogenase activity

A mixture of 50 mM Tris–HCl buffer (pH 7.0), 0.1 mM NAD+, and 0.3 mM P5C was used in the P5CDH reaction. An enzymatic extract of 0.2 mL was added to a final volume of 2.0 mL to start the reaction. An enzyme extract-free blank was prepared from the reaction mixture. A linear decrease in absorbance at 340 nm was observed after mixing for 5 minutes, and enzyme activity was measured after 2 minutes at 30°C. The molar extinction coefficient of NAD(P)H was used to quantify P5CDH activity and expressed as nmol NADH formed mg^−1^ protein min^−1.^

### Proline dehydrogenase activity

ProDH enzyme extract was incubated at 28°C in a reaction buffer containing 100 mM Na_2_CO_3_-NaHCO_3_ (pH 10.3), 20 mM L-proline, and 10 mM NAD^+^ to determine its activity, and then ProDH dependent NAD^+^ reduction was measured at 340 nm. The quantity of enzyme catalyzing the synthesis of 1 μmol of NADH per minute is defined as one unit of ProDH activity.

### Protein estimation

The protein content of the plants was determined according to Bradford (Bradford, 1976), using Bovine serum albumin (BSA) as a standard.

### Statistical analysis

All experiments were conducted in triplicates (n = 3), except for FM, BA, and RWC where 10 replicates were used. Using GraphPad Prism 8, two-way ANOVA was carried out and the results in the graphs were given as arithmetic mean ± standard error (SE). Tukey’s post-hoc test was employed for identifying statistical differences at the 0.05 probability level.

## Results

### Growth and tolerance index of salt-stressed and Zn treated plants

**Figure 1:**
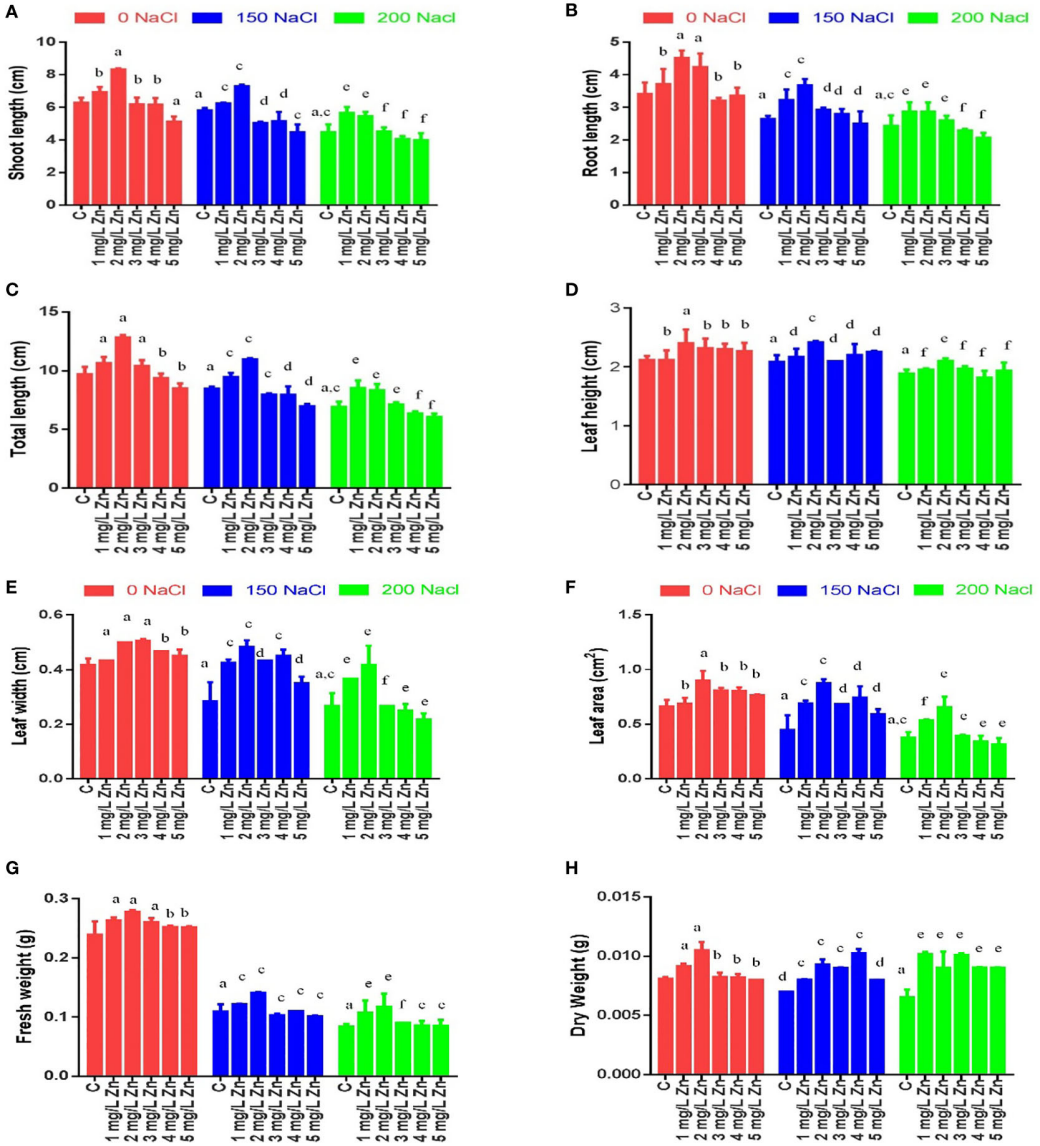
Effects of Nacl and Zn on morphological parameters: **(A)** Shoot length **(B)** Root length **(C)** Total length **(D)** Leaf height **(E)** Leaf width **(F)** Leaf area **(G)** Fresh weight and **(H)** dry weight. The different letters on bars represent the significant differences (a, significant; b, non-significant compared to control; c, significant; d, non-significant compared to 150 mM NaCl and e, significant; f, non-significant compared to 200 mM at p ≤ 0.05.

Morphological parameters were measured after 22 days of sowing, and it was observed that the shoot length (SL) decreased significantly under salt stress in a dose-dependent manner. As can be seen from **Figure 1A** maximum decrease in SL was reported at 200mM NaCl (28.84%), while as a decrease of 7.43% in SL was observed at 150mM NaCl in comparison to control. However, the exogenous application of Zn was not only seen to alleviate the negative effects of salt on SL, but also increase the SL under normal conditions. In comparison to control the low doses of Zn (1 mg/L and 2mg/L) were seen to be more efficient in terms of salt stress alleviation by showing an increase in SL by 10.44% and 32.38% respectively. However, the higher doses of Zn (3, 4, 5 mg/L) were seen to be toxic as evidenced by a decrease in SL by 1.59%, 1.76%, and 18.4%, respectively. In salt-treated plants, the low doses of Zn rescued the negative impact as the SL increased by 7.26% and 25.5% in treatments 150 mM NaCl +1 mg/L Zn and 150 mM NaCl +2 mg/L Zn treated plants respectively in comparison to plants treated only with 150 mM NaCl. Similarly, the treatments 200 mM NaCl, + 1 mg/L Zn, 2mg/L were also reported to be effective in rescuing salt damage on SL (**Figure 1A**).

The root length (RL) also decreased at 200mM NaCl (28.66%), while as a decrease of 22.47% in RL was observed at 150mM NaCl in comparison to control (no salt/Zn treatment). However, the exogenous application of Zn alleviated the negative effects of salt on RL. We report an 8.79%, 32.24% and 24.1% increase in RL at 1 mg/L, 2mg/L and 3mg/L Zn respectively in comparison to control plants. The higher doses of Zn (4, 5 mg/L) were seen to be toxic as there was a decrease in RL at these concentrations by 6.1% and 1.6% respectively in comparison to control. In salt-treated plants, Zn rescued the negative impact of salt on RL, and we observed an increase of 21.84%, 39.07%, 10.5% and 5.88% in 150 mM NaCl+1 mg/L, 150 mM NaCl +2mg/L, 150 mM NaCl+3 mg/L and 150 mM NaCl+4 mg/L treated plants respectively in comparison to plants treated only with 150 mM NaCl. However, RL decreased by 5.4% in plants treated with 150 mM NaCl +4 mg/L treated plants as compared to 150 mM NaCl. Similarly, at 200 mM NaCl, a low dose (1 mg/L, 2mg/L, and 3mg/L) of Zn was found to be effective in rescuing salt damage. An increasing in RL by 17.8%, 17.8% and 6.84% at 1 mg/L, 2mg/L, and 3mg/L was observed however application of 4 mg/L and 5 mg/L Zn decreased RL when compared to plants treated with 200mM salt (**Figure 1B**).

The total length (TL) in proso millet under salt stress decreased by 12.72% at 150mM NaCl and by 28.78% at 200 mM NaCl. We report a 9.86%, 32.33% and 7.45% increase in TL at 1 mg/L, 2mg/L and 3mg/L Zn respectively in comparison to control plants. The higher doses of Zn (4, 5 mg/L) were seen to be toxic as there was a decrease in TL at these concentrations by 3.32% and 12.5% respectively in comparison to control. In salt-treated plants, Zn mitigated the negative impact of salt on TL, and we observed an increase of 11.82% and 29.7% in 150 mM NaCl+1 mg/L, 150 mM NaCl+ 2mg/L treated plants respectively in comparison to plants treated only with 150 mM NaCl. However, TL decreased by 5.9%, 6% and 17.6% in plants treated with 150 mM NaCl+3 mg/L, 150 mM NaCl+4 mg/L and 150 mM NaCl +5 mg/L respectively as compared to 150 mM NaCl. Similarly, at 200 mM NaCl, low doses (1 mg/L, 2mg/L, and 3mg/L) of Zn were found to be effective in rescuing salt damage. An increase in TL by 23.5%, 20.6% and 3.22% at 200 mM NaCl+1 mg/L, 200 mM NaCl+2mg/L, and 200 mM NaCl +3mg/L was observed however application of 200 mM NaCl+4 mg/L and 200 mM NaCl+5 mg/L Zn decreased TL when compared to plants treated with 200 mM NaCl (**Figure 1C**).

The leaf height (LH) in proso millet under salt stress decreased by 1.57% at 150mM NaCl and by 11.02% at 200 mM NaCl. However, the application of 1 mg/L Zn does not affect LH. We found 13.39%, 9.45%, 8.66% and 7.09% increase in LH at 2mg/L, 3mg/L, 4 mg/L and 5 mg/L Zn respectively in comparison to control plants. In salt-treated plants, Zn mitigated the negative impact of salt on LH, and we observed an increase of 4%, 16%, 0.8%, 5.6% and 8% in 150 mM NaCl+1 mg/L, 150 mM NaCl+2mg/L, 150 mM NaCl+3mg/L, 150 mM NaCl+4mg/L and 150 mM NaCl+5mg/L treated plants respectively in comparison to plants treated only with 150 mM NaCl. Similarly, at 200 mM NaCl, (1 mg/L, 2mg/L, and 3mg/L) of Zn were found to be effective in rescuing salt damage. An increasing in LH by 3.54%, 11.5% and 4.42% at 1 mg/L, 2mg/L, and 3mg/L when compared to plants treated with 200 mM NaCl (**Figure 1D**).

The leaf width (LW) in proso millet under salt stress decreased by 32% at 150mM NaCl and by 36% at 200mM NaCl. However, after the application Zn we found 4%, 20%, 12% and 12% and 8% increase in LW at 1mg/L, 2mg/L, 3mg/L, 4 mg/L and 5 mg/L Zn respectively in comparison to control plants. In salt-treated plants, Zn mitigated the negative impact of salt on LW, and we observed an increase of 50%, 70.5%, 52.9%, 58.8% and 23.58% in 150 mM NaCl+1 mg/L, 150 mM NaCl+2mg/L, 150 mM NaCl+3mg/L, 150 mM NaCl +4mg/L and 150 mM NaCl+ 5mg/L treated plants respectively in comparison to plants treated only with 150 mM NaCl. Similarly, at 200 mM NaCl, low levels of Zn (1 mg/L and 2mg/L) were found to be effective in rescuing salt damage. An increasing in LW by 37.5% and 56.25% was found at 1 mg/L and 2mg/L, whereas at 3mg/L no change in LW was observed when compared to plants treated with 200 mM NaCl. However, higher concentrations (4 mg/L and 5mg/L) of Zn showed a toxic effect and decreased LW (**Figure 1E**).

The leaf area (LA) in proso millet under salt stress decreased by 32.66% at 150mM NaCl and by 43.3% at 200mM NaCl. However, after the application of Zn we found 4%, 35.9%, 22% and 21% and 15.35% increase in LA at 1mg/L, 2mg/L, 3mg/L, 4 mg/L and 5 mg/L Zn respectively in comparison to control plants. In salt-treated plants, Zn mitigated the negative impact of salt on LA, and we observed an increase of 54.76%, 96.44.5%, 53.08%, 66.91% and 32.52% in 150 mM NaCl+1 mg/L, 150 mM NaCl+2mg/L, 150 mM NaCl+3mg/L, 150 mM NaCl+4mg/L and 150 mM NaCl+5mg/L treated plants respectively in comparison to plants treated only with 150 mM NaCl. Similarly, at 200 mM NaCl, low levels of Zn (1 mg/L, 2mg/L and 3mg/L) were found to be effective in rescuing salt damage. An increase in LA by 42.8%, 74.47% and 4.77% was found at 200 mM NaCl+1 mg/L, 200 mM NaCl + 2mg/L and 200 mM NaCl +3mg/L when compared to plants treated with 200 mM NaCl. However, higher concentrations (4 mg/L and 5mg/L) of Zn showed a toxic effect by decreasing LA (**Figure 1F**).

Tolerance index (TI) improved in the plants treated with Zn in comparison to NaCl treatments only. The TI in shoots decreased by 4.91% at 150 mM NaCl and 19.12% at 200 mM NaCl concerning control. However, after the application of Zn we found 8.19% and 37.7% increase in TI at 1mg/L and 2mg/L, respectively in comparison to control plants. It was also seen that higher doses of Zn proved toxic as they decreased TI. In salt-treated plants, Zn mitigated the negative impact of NaCl, and we observed an increase of 8.04% and 24.7% in 150 mM NaCl +1 mg/L and 150 mM NaCl + 2mg/L respectively in comparison to plants treated only with 150 mM NaCl. The higher doses (150 mM NaCl+3mg/L, 150 mM NaCl+4mg/L and 150 mM NaCl+5mg/L) proved toxic as they decreased TI by 12%, 0.5% and 14.9% respectively in comparison to plants treated only with 150 mM NaCl. Similarly, at 200 mM NaCl, low levels of Zn (1 mg/L and 2mg/L) were found to be effective in rescuing salt damage. An increase in TI by 22.9% and 5.4% were found at 200 mM NaCl+1 mg/L and 200 mM NaCl+2mg/L when compared to plants treated with 200mM salt. However, higher concentrations of Zn showed toxic effect by decreasing TI (**Table 1**).

**Table 1:** Effect of Zn and collective effect of salt and Zn on root tolerance index (root TI %), shoot tolerance index (shoot TI %) and RWC of proso millet (PM).

Parameter	Treatments														
	C	1mg/L Zn		2mg/L Zn	3mg/L Zn			4mg/L Zn				5mg/L Zn	150mM NaCl	150mM NaCl+1mg/L Zn	150mM NaCl+2mg/L Zn
TI % (Root)	99.5 ± 0.7	103.9 ± 1.34^a^		129.7 ± 0.98^a^	135.63 ± 0.90^a^			97.02 ± 0.041^a^				95.53 ± 0.8^a^	74.05 ± 0.64^a^	104.94 ± 1.33^c^	111.87 ± 1.23^c^
TI % (Shoot)	100 ± 0	107.59 ± 0.84^a^		136.85 ± 1.2^a^	94.54 ± 0.76^a^			99.5 ± 0.70^a^				89.53 ± 0.7^a^	94.54 ± 0.76^a^	101.86 ± 1.22^c^	118.03 ± 0.76^c^
RWC (%)	96.4 ± 0.008	96.47 ± 0.097^a^		96.56364 ± 0.2^a^	96.63 ± 0.32^a^			96.6 ± 0.28^a^				96.6 ± 0.27^a^	93 ± 0^a^	93.16 ± 0.23^d^	93.33 ± 0.46^c^
Parameter	Treatments														
	150mM NaCl+3mg/L Zn		150mM NaCl+4mg/L Zn	150mM NaCl+5mg/L Zn		200mM NaCl			200 mM NaCl+1mg/L Zn		200 mM NaCl+2mg/L Zn		200 mM NaCl+3 mg/L Zn	200 mM NaCl+4mg/L Zn	200 mM NaCl+5mg/L Zn
TI % (Root)	83.90 ± 0.57^c^		86.13 ± 0.19^c^	65.83 ± 1.17^c^		62.37 ± 0.52^a,c^			93.55 ± 0.79^c^		75.24 ± 0.34^c^		78.11 ± 0.44^c^	67.83 ± 1.15^c^	55.44 ± 0.62^c^
TI % (Shoot)	83.05 ± 0.78^c^		94.01 ± 0.73^c^	80.43 ± 0.61^c^		80.43 ± 0.61^a,c^			98.72 ± 1.02^c^		85.12 ± 0.17^c^		74.97 ± 1.38^c^	69.19 ± 0.28^c^	72.33 ± 0.47^c^
RWC (%)	92.04 ± 1.35^c^		91.95 ± 1.47^c^	94.4 ± 3^c^		91.3 ± 0.003^a^			91.55 ± 0.35^f^		91.890.83^e^		90.11 ± 1.6^e^	90.02 ± 1.8^e^	89.88 ± 2.00^e^

Values represent the % change with respect to control. The different letters on bars represent the significane.

The TI in roots also decreased by 25.49% at 150mM NaCl and 37.25% at 200mM NaCl with respect to control. However, after the application of Zn we found 4.9%, 30.3% and 36.27% increase in TI of root at 1mg/L, 2mg/L and 3mg/L Zn, respectively in comparison to control plants. However higher doses of Zn proved toxic as they decreased TI. In salt-treated plants, Zn mitigated the negative impact of NaCl, and we observed an increase of 42.1%, 51.3%, 13.15% and 15.78% in 150 mM NaCl+1 mg/L, 150 mM NaCl+2mg/L, 150 mM NaCl+3mg/L and 150 mM NaCl+4mg/L respectively in comparison to plants treated only with 150 mM NaCl. The higher doses (150 mM NaCl+5mg/L) proved toxic as they decreased TI by 10.52% respectively in comparison to plants treated only with 150 mM NaCl. An increasing in TI by 50%, 20.31%, 25% and 9.37 were found at 200 mM NaCl+1 mg/L, 200 mM NaCl+2mg/L, 200 mM NaCl +3mg/L and 200 mM NaCl+4mg/L when compared to plants treated with 200 mM NaCl. However, higher concentrations of Zn showed a toxic effect by decreasing TI (**Table 1**).

The fresh weight (FW) of proso millet decreased by 55.15% at 150mM NaCl and 65% at 200 mM NaCl with respect to control. However, after the application of Zn we found a 16.5%, 23.3% and 14.34% increase in FW at 1mg/L, 2mg/L and 3mg/L Zn, respectively in comparison to control plants. However higher doses of Zn (4mg/L and 5mg/L Zn) increased it by 12.1% each. In NaCl treated plants, Zn mitigated the negative impact of NaCl, and we observed an increase of 20% and 42% in 150 mM NaCl+1 mg/L and 2mg/L+150 mM NaCl treated plants respectively in comparison to plants treated only with 150 mM NaCl. An increasing in FW by 51.5%, 65.2% and 12.04% were found at 1 mg/L+200 mM NaCl, 2mg/L+200 mM NaCl and 3mg/L+200 mM NaCl treated plants in comparison to 200 mM NaCl. However higher doses prove toxic (**Figure 1G**).

A similar trend was observed in the case of dry weight (DW) of proso millet, as DW decreased by 12.5% at 150 mM NaCl and 12.5% at 200 mM NaCl with respect to control. However, after the application of Zn, we found a 12.5% increase in DW at 1mg/L and 2mg/L Zn, respectively in comparison to control plants. It was seen that higher doses of Zn did not affect DW. In salt-treated plants, Zn mitigated the negative impact of NaCl, and we observed that all doses applied, increased DW with a maximum increase of 42.85% in 4mg/L+150 mM NaCl treated plants in comparison to plants treated only with 150 mM NaCl. All the doses of Zn increased DW and maximum increasing of 42.8% was found in 1mg/L+200 mM NaCl to 3mg/L+200 mM NaCl treated plants in comparison to 200mM salt (**Figure 1H**).

RWC decreased noticeably as NaCl treatments increased. In proso millet, the decrease in RWC was 3.52–5.3% at 150–200 mM with respect to control. However, after the application Zn we found that all the doses increased RWC with a maximum increase of 0.48% at 3mg/L Zn with respect to control plants. In NaCl-treated plants, Zn mitigated the negative impact of salt, and we observed that increased RWC of 0.35% and 0.711% at 1mg/L+150 mM NaCl and 2mg/L+150 mM NaCl treated plants in comparison to plants treated only with 150 mM NaCl. Higher doses (3mg/L+150 mM NaCl, 4mg/L+150 mM NaCl, and 5mg/L+150 mM NaCl) showed toxic effect as they reduced RWC in comparison to plants treated only with 150 mM NaCl. An increase in RWC by 0.55% and 1.29% at 1 mg/L+200 mM NaCl and 2mg/L+200 mM NaCl was observed in comparison to plants treated with 200mM salt (**Table 1**).

### Effect on membrane stability index and electrolyte leakage

**Figure 2:**
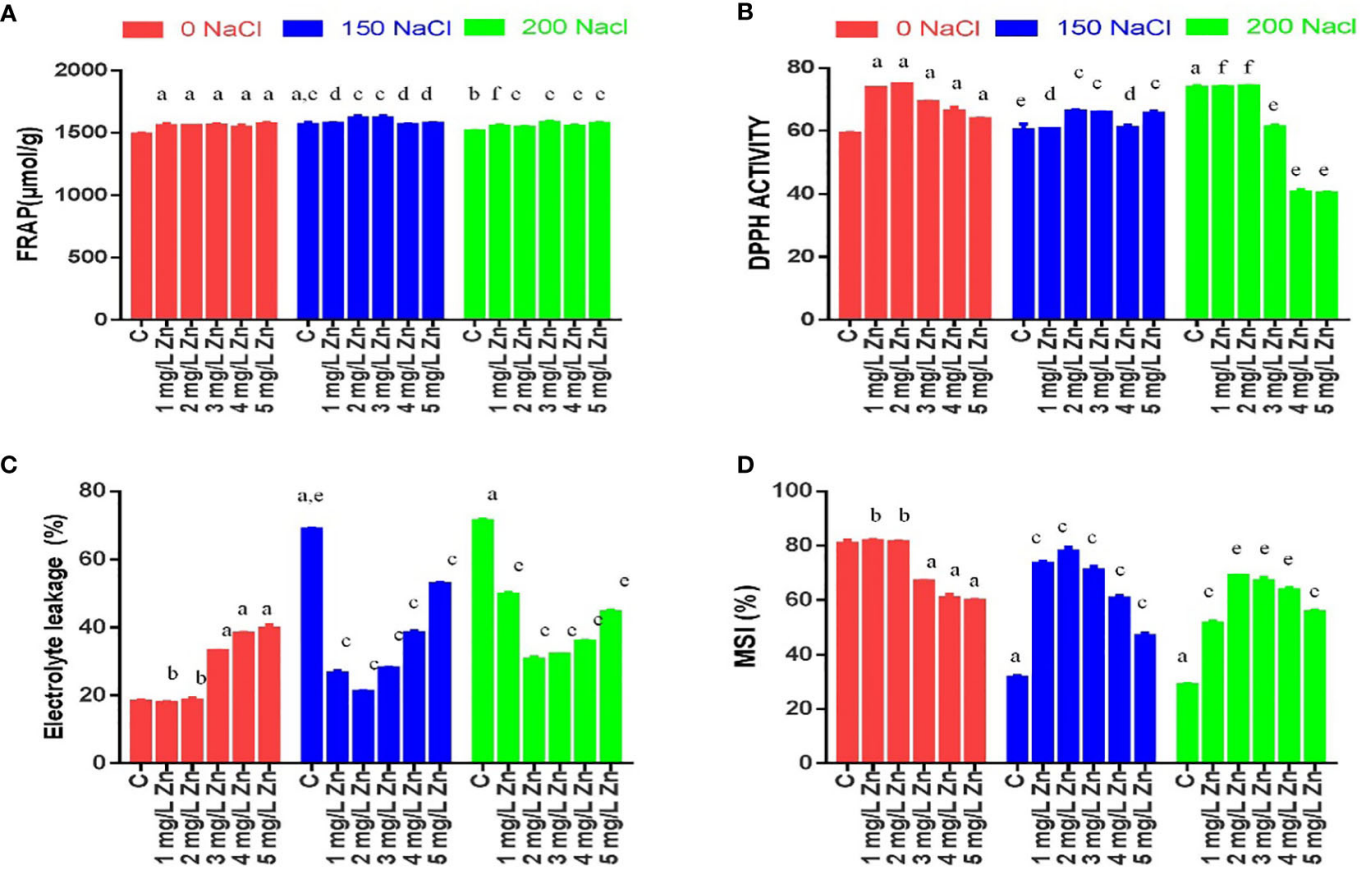
Effects of Nacl and Zn on membrane stability and antioxidant potential: **(A)** FRAP **(B)** DPPH **(C)** Electrolyte leakage **(D)** Membrane stability index. Thedifferent letters on bars represent the significant differences (a, significant; b, non-significant compared to control; c, significant; d, non-significantcompared to 150 mM NaCl and e, significant; f, non-significant compared to 200 mM at p ≤ 0.05.

The MSI is an important feature that measures the influence of stress on cell membrane electrolyte conductivity. In general, a higher MSI indicates greater tolerance to salt stress. **Figure 2D** shows that with the increase in salt stress, MSI reduces considerably. The decrease in MSI from 61.5% to 65.04% at 150–200 mM NaCl was observed with respect to control. However, after the application Zn we found that MSI increased. The MSI after the application Zn were increased by 1.38% and 0.34% at 1mg/L and 2mg/L, Zn respectively in comparison to control plants. The higher doses of Zn (3mg/L, 4 mg/L and 5 mg/L) were toxic as they decreased MSI by 17.99%, 24.4% and 26.9% with respect to control plants. In salt treated plants, Zn mitigated the negative impact of salt as it increased MSI by 132.57%, 151.58%, 129.56%, 95.47% and 51.6% in 1 mg/L+150 mM NaCl, 2mg/L+150 mM NaCl, 3mg/L+150 mM NaCl, 4mg/L+150 mM NaCl and 5mg/L+150 mM NaCl treated plants respectively in comparison to plants treated only with 150 mM NaCl. Similarly, an increase in MSI by all the doses of Zn was found when compared to plants treated with 200mM salt. The maximum increase of 141.98% in MSI was at 2 mg/L +200NaCl.

EL is dependent on MSI and is inversely proportional to it, so an increase in salt stress increased the EL. The increase in EL from 271.94% to 287.29% at 150–200 mM NaCl was observed with respect to control. However, after the application Zn we found that EL decreased. The EL after the application of Zn were decreased by 6.1% and 1.51% at 1mg/L and 2mg/L, Zn respectively in comparison to control plants. The higher doses of Zn (3mg/L, 4 mg/L and 5 mg/L) were toxic as they increased EL by 79.4%, 107.84% and 118.97% with respect to control plants. In salt-treated plants, Zn mitigated the negative impact of salt as it decreased EL by 60.6%, 69.16%, 59.12%, 43.56% and 23.54% in 1 mg/L+150 mM NaCl, 2mg/L+150 mM NaCl, 3mg/L+150 mM NaCl, 4mg/L+150 mM NaCl and 5mg/L+150 mM NaCl treated plants respectively in comparison to plants treated only with 150 mM NaCl. Similarly, a decrease in EL by all the doses of Zn was found when compared to plants treated with 200mM salt. The maximum decrease of 56.6% in EL was at 2 mg/L +200 NaCl (**Figure 2C**).

### Biochemical effects of salinity and zinc on total chlorophyll, chlorophyll a and chlorophyll b

**Figure 3:**
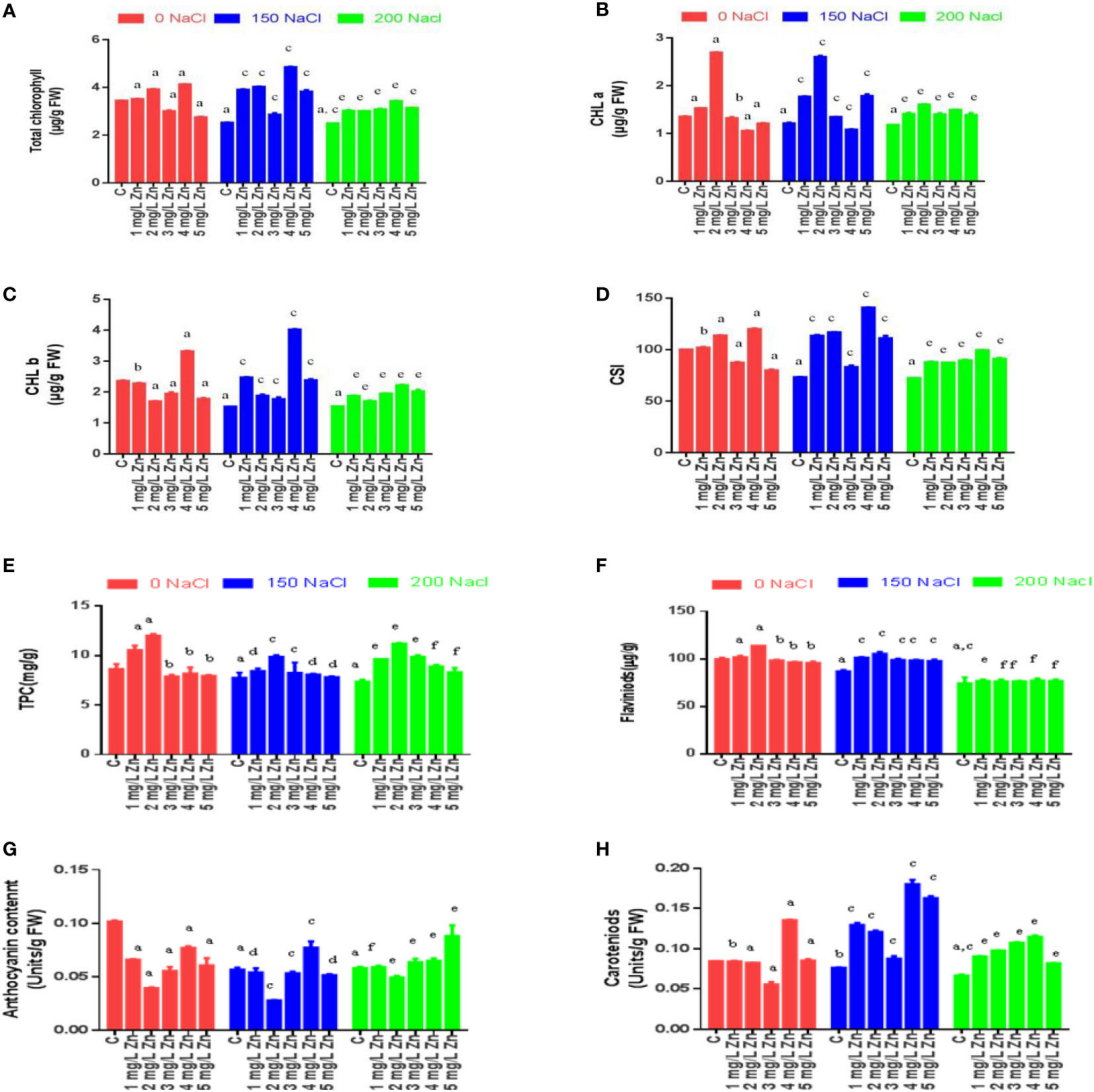
Effects of Nacl and Zn on photosynthetic pigments, chlorophyll stability index and total phenolic contents: **(A)** Total chlorophyll **(B)** Chlorophyll a **(C)** Chlorophyll b **(D)** Chlorophyll stability index **(E)** Total phenolic content **(F)** Flavonoids **(G)** Anthocyanin **(H)** Caroteniods. The different letters on bars represent the significant differences (a, significant; b, non-significant compared to control; c, significant, d = non-significant compared to 150 mM NaCl and e, significant; f, non-significant compared to 200 mM at p ≤ 0.05.

The total chlorophyll content (TCC), chlorophyll a (Chl a) and chlorophyll b (Chl b) concentrations were affected by Zn, NaCl and Zn with NaCl treatments (**Figure 3**). The levels of total chlorophyll, chlorophyll a, chlorophyll b in the proso millet leaves were significantly reduced with the rising salinity levels. The TCC decreased at 200 mM NaCl (27.56%), while, a decrease of 26.6% was observed at 150 mM NaCl in comparison to control (no salt/Zn treatment). However, the exogenous application of Zn alleviated the negative effects of salt. We report a 1.9%, 13.7% and 20.0% increase in TCC at 1 mg/L, 2mg/L and 4mg/L Zn respectively in comparison to control plants. The higher doses of Zn (5 mg/L) were seen to be toxic as there was a decrease in TCC by 20.06% in comparison to the control. In NaCl-treated plants, Zn mitigated the negative impact of NaCl on TCC, and we observed an increase of TCC at all Zn concentrations with maximum increases of 91.93% at 4 mg/L+150 mM NaCl treated plants in comparison to plants treated only with 150 mM NaCl. Similarly, all the doses of Zn increased TCC at 200 mM NaCl and the maximum increase of 37.2% at 4 mg/L was observed in comparison to plants treated with 200 mM NaCl. The Chl a decreased at 150mM NaCl (10.4%) and 200mM NaCl (12.55%) and in comparison to control (no salt/Zn treatment). However, the exogenous application of Zn alleviated the negative effects of salt. We report a 13% and 99% increase in Chl a at 1 mg/L and 2 mg/L Zn respectively in comparison to control plants. The higher doses of Zn decreased Chl a in comparison to the control. In NaCl-treated plants, Zn mitigated the negative impact of NaCl on Chl a, and we observed an increase of Chl a at 1 mg/L, 2mg/L Zn, 3 mg/L and 5mg/L Zn concentrations with maximum increases of 114.85% at 150 mM NaCl+2 mg/L treated plants in comparison to plants treated only with 150 mM NaCl. Similarly, all the doses of Zn increased Chl a at 200mM level and the maximum increase of 35.5% at 2 mg/L was observed in comparison to plants treated with 200 mM NaCl. The Chl b decreased at 150mM NaCl (35.36%) and 200mM NaCl (34.9%) in comparison to control (no salt/Zn treatment). We report a 40.5% increase in Chl b at 4mg/L Zn respectively in comparison to control plants. In salt-treated plants, Zn mitigated the negative impact of salt on Chl b, and we observed an increase of Chl b at all the concentrations with maximum increases of 163.86% at 4 mg/L+150 mM NaCl treated plants in comparison to plants treated only with 150 mM NaCl. Similarly, all the doses of Zn increased Chl b at 200 mM level and the maximum increase of 44.42% at 4 mg/L was observed in comparison to plants treated with 200 mM NaCl (**Figure 3C**).

### Effects of salinity and zinc on carotenoids, anthocyanin, total phenolic content, flavonoids and chlorophyll stability index

Like chlorophyll, carotenoid concentration was also reduced following salt treatments, with a drop of 9.5-21.19%. at 150–200 mM NaCl with reference to control. The supplementation of 3 mg/L and 4 mg/L Zn increased carotenoids by 60.75% and 1.33% respectively with reference to control. Furthermore, when Zn was administered with NaCl treatments, carotenoid concentration improved with a maximum increase of 136.10% at 4 mg/L Zn with reference to 150 mM NaCl and an increase of 72.50% at 4 mg/L Zn with reference to 200 mM NaCl treated plants (**Figure 3H**).

Anthocyanin content was also reduced following salt treatments, with a drop of 44.06-42.88% at 150–200 mM NaCl respectively with reference to control. However, the supplementation of Zn alone decreased anthocyanin content with reference to control. Moreover, when Zn was administered with NaCl treatments, anthocyanin concentration improved by 36.17% only at 4 mg/L Zn with reference to 150 mM NaCl and an increase of 52.06% at 5 mg/L Zn with reference to 200 mM NaCl treated plants (**Figure 3G**).

Total phenolic content (TPC) was also reduced following NaCl treatments, with a drop of 10.14-14.49% at 150–200 mM NaCl respectively with reference to control. However, the supplementation of Zn increased TPC content by 22.46% and 39.13% at 1 mg/L and 2 mg/L Zn with reference to control. However higher doses of Zn proved toxic and reduced TPC with reference to control. When Zn was administered with salt treatments, TPC improved at all concentrations and the maximum increase of 27.4% at 2 mg/L Zn with reference to 150 mM NaCl and of 51.6% at 2 mg/L Zn with reference to 200 mM NaCl treated plants (**Figure 3E**).

Flavonoid content (FC) also reduced following salt treatments, with a drop of 13.6-25.5% at 150–200 mM NaCl respectively with reference to control. However, the supplementation of Zn increased FC content by 2% and 14% at 1 mg/L and 2 mg/L Zn with reference to control. However higher doses of Zn proved toxic and reduced FC with reference to control. When Zn was administered with salt treatments, FC improved at all concentrations and the maximum increase of 21.26% at 2 mg/L Zn with reference to 150 mM NaCl and 4.02% at 4 mg/L Zn with reference to 200 mM NaCl treated plants (**Figure 3F**).

The chlorophyll stability index (CSI) is a cardinal aspect that determines the photosynthetic ability of a plant. **Figure 3D** that with the increase in salt CSI reduces considerably. The decrease in CSI from 26.6% to 27.56% at 150–200 mM NaCl was observed with respect to control. However, after the application Zn we found that CSI increased. The CSI after the application of Zn were increased by 2% and 13.7% at 1mg/L and 2mg/L Zn respectively in comparison to control plants. The higher doses of Zn (3mg/L, 4 mg/L and 5 mg/L) were toxic as they decreased CSI. In salt treated plants, Zn mitigated the negative impact of salt as it increased CSI by 54.6%, 59.24%, 13.22%, 92% and 11.06% in 1 mg/L+150 mM NaCl, 2mg/L+150 mM NaCl, 3mg/L+150 mM NaCl, 4mg/L+150 mM NaCl and 5mg/L+150 mM NaCl treated plants respectively in comparison to plants treated only with 150 mM NaCl. Similarly, an increase in CSI by all the doses of Zn was found when compared to plants treated with 200 mM NaCl. The maximum increase of 37.2% in CSI was at 4 mg/L +200 NaCl.

### DPPH and FRAP activities in response to salinity and zinc

In response to salinity, the DPPH antioxidant capacity of leaf extracts, increased by 2.05% at 150mM NaCl and by 24.76% at 200 mM NaCl with reference to control plants. All the doses of Zn increased DPPH activity and the maximum increase of 26.5% was observed at 2 mg/L Zn with reference to control plants. However, when Zn was administered with salt treatments, it increased DPPH activity and a maximum increase of 9.65% at 2 mg/L Zn with reference to 150 mM NaCl. An increasing in DPPH activity by 0.62% was found at 2 mg/L+200 mM NaCl treated plants in comparison to 200 mM NaCl. However higher doses prove toxic (**Figure 2B**).

Similar results were observed for FRAP antioxidant capacity. FRAP activity increased by 5.4% at 150 mM NaCl and by 2.54% at 200 mM NaCl with reference to control plants. All the doses of Zn increased FRAP activity and the maximum increase of 6.12% was observed at 5 mg/L Zn with reference to control plants. However, when Zn was administered with salt treatments, it increased FRAP activity and a maximum increase of 3.43% at 2mg/L and 3mg/L Zn with reference to 150 mM NaCl. All the concentrations of Zn increased FRAP activity and the maximum increase of 4.21% was found at 3mg/L+200 mM NaCl treated plants in comparison to 200 mM NaCl (**Figure 2A**).

### Effects of salinity and zinc on proline and enzymes of proline pathway

**Figure 4:**
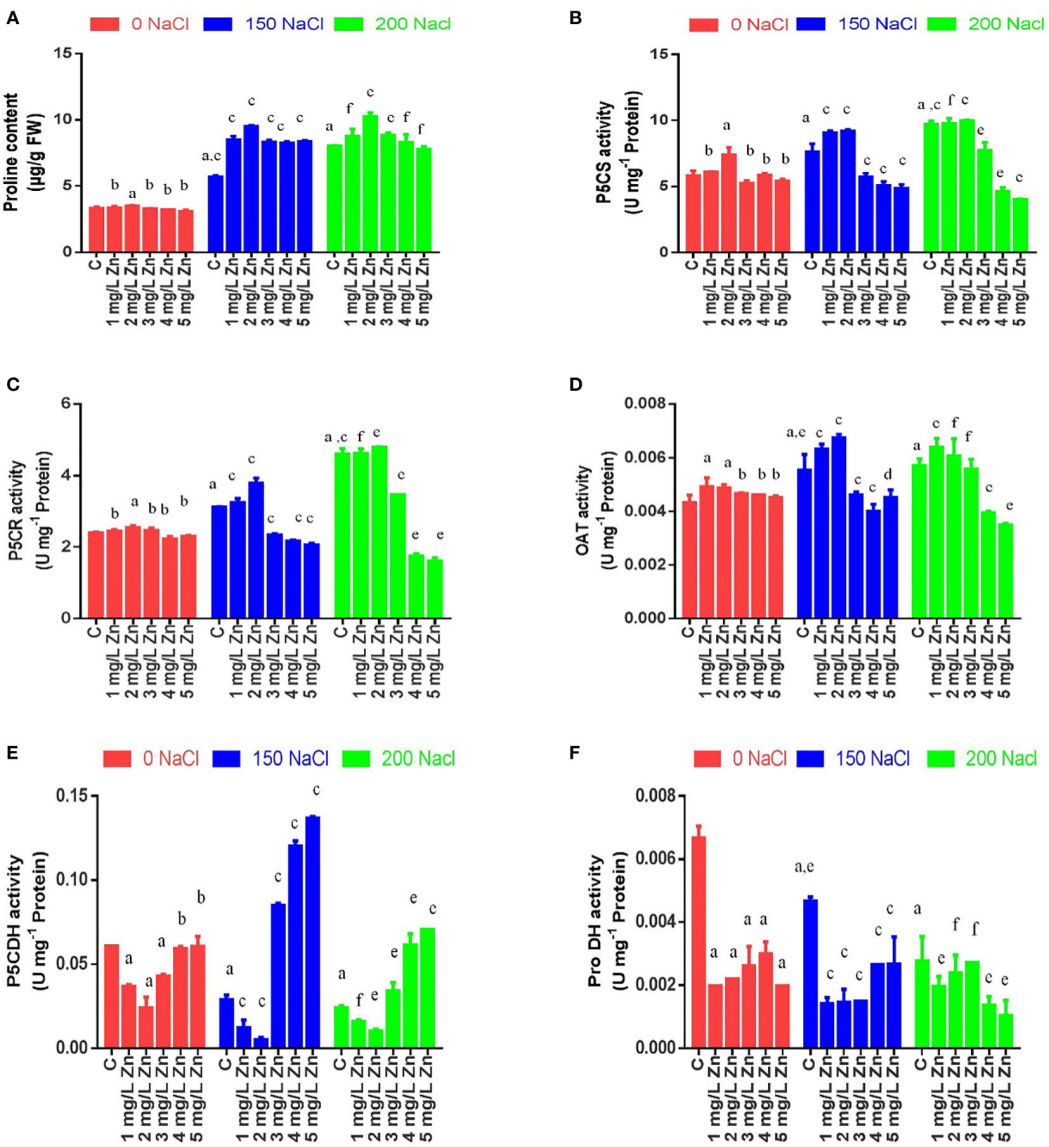
Effects of Nacl and Zn on total proline content and enzymes activities related to proline biosynthetic pathway: **(A)** Proline content **(B)** P5CS activity **(C)** P5CR activity **(D)** OAT activity **(E)** P5CDH activity **(F)** ProDH activity The different letters on bars represent the significant differences (a. significant; b. non-significant compared to control; c, significant; d, non-significant compared to 150 mM NaCl and e, significant; f, non-significant compared to 200 mM at p ≤ 0.05.

The proline content (PC) in proso millet under salt stress increased by 71.65% at 150 mM NaCl and 141.73% at 200 mM NaCl. However, the supplementation of Zn further increased PC content by 1.18% and 5.54% at 1 mg/L and 2 mg/L Zn with reference to control. However higher doses of Zn proved toxic and reduced PC with reference to control. When Zn was administered with salt treatments, PC improved at all concentrations and the maximum increase of 66.65% at 2 mg/L Zn with reference to 150 mM NaCl and 27.5% at 2 mg/L Zn with reference to 200 mM NaCl treated plants (**Figure 4A**).

P5CS activity in proso millet under salt stress increased by 30% at 150 mM NaCl and increased by 66.6% at 200 mM NaCl. The application of Zn (1 mg/L and 2 mg/L) had a positive impact on the activity of P5CS as it increased by 4.66% and 26.96%, whereas the application of higher doses of Zn (3 mg/L, 4 mg/L and 5 mg/L) decreased it in comparison to control. In salt treated plants, a lower concentration of Zn further increased the activity of P5CS by 19.344% and 21% at 150 mM NaCl+1 mg/Land 2mg/L+150 mM NaCl treated plants respectively in comparison to plants treated only with 150 mM NaCl. However higher doses of Zn (3 mg/L, 4 mg/L and 5 mg/L) decreased it in comparison to 150 mM NaCl. An increase in P5CS activity by 0.64% and 3% were found at 1 mg/L+200 mM NaCl and 2mg/L+200 mM NaCl treated plants respectively in comparison to 200 mM NaCl. However higher doses prove toxic in comparison to 200 mM NaCl (**Figure 4B**).

Similar results were observed for P5CR activity. P5CR activity in under salt stress increased by 29.5% at 150 mM NaCl and 91.3% at 200 mM NaCl. However, the supplementation of Zn increased P5CR activity by 2.01%, 5.86 and 2.42% at 1 mg/L, 2 mg/L and 3 mg/L Zn respectively with reference to control. It was seen that higher doses of Zn proved toxic and reduced P5CR activity with reference to control. When Zn was administered with salt treatments, P5CR activity improved at lower concentrations (1 mg/L and 2 mg/L) and the maximum increase of 21.66% at 2 mg/L Zn with reference to 150 mM NaCl and 4% at 2 mg/L Zn with reference to 200 mM NaCl treated plants was observed. However higher doses of Zn proved toxic and reduced P5CR activity (**Figure 4C**).

The activity of OAT also increased under salt stress by 27.85% at 150mM NaCl and 32% at 200 mM NaCl. However, all the doses of Zn increased OAT activity and the maximum increase of 13.65% was observed at 1 mg/L Zn with reference to control. When Zn was administered with salt treatments, OAT activity improved at lower concentrations (1 mg/L and 2 mg/L) and the maximum increase of 21.84% at 2 mg/L Zn with reference to 150 mM NaCl and 11.73% at 1 mg/L Zn with reference to 200 mM NaCl treated plants was observed. However higher doses of Zn reduced OAT activity (**Figure 4D**).

The activity of PDH activity decreased under salt stress by 33.5% at 150 mM NaCl and 58.48% at 200 mM NaCl. However, all the doses of Zn decreased PDH activity and the maximum decrease of 70.38% was observed at 1 mg/L Zn with reference to control. When Zn was administered with salt treatments, PDH activity further decreased at all concentrations and the maximum decrease of 67.09% at 2 mg/L Zn with reference to 150 mM NaCl and 62.51% at 5 mg/L Zn with reference to 200 mM NaCl treated plants was observed. The activity of P5CDH activity decreased under salt stress by 52.65% at 150mM NaCl and 60.68% at 200mM NaCl. However, all the doses of Zn decreased P5CDH activity and the maximum decrease of 61% was observed at 2 mg/L Zn with reference to control. When Zn was applied with salt, it decreased the activity of P5CDH by 57.5% and 82.5% at 1 mg/L and 2 mg/L Zn respectively in comparison to 150 mM NaCl. The decrease in P5CDH activity by 33.7% and 56.7% at 1 mg/L and 2mg/L was observed, however application of 3 mg/L, 4 mg/L and 5 mg/L Zn increased P5CDH when compared to plants treated with 200 mM NaCl (**Figures 4E**, **F**).

## Discussion

Salt stress has negative consequences on plant growth and plants respond to this by accumulating wide range of metabolic products, principally, amino acids accumulate in plants, which are fundamental to plant developmental processes. There is an optimistic relationship between proline amassing and stress in plants which points towards its role in stress mitigation by osmotic adjustments. The present study aimed to study the role of Zn in stimulating proline metabolism and stress-responsive elements. To assess plant salt tolerance in proso millet, the morphological features were studied. The results of our study indicate that NaCl (150mM, 200mM NaCl) has a negative impact on growth and development in proso millet and the maximum damage was observed at higher salt levels. The results were in agreement with previous studies which indicated that salinity decreases growth, SL, RL, DW, FW, LW, and LA as observed in millets viz., *Pennisetum glaucum* L., *Eleusine coracana* L., *Setaria italica* L. and *Paspalum scrobiculatum* L. (Khushdil et al., 2019; Kothai and Roselin Roobavathi, 2020; Mukami et al., 2020; Rathinapriya et al., 2020; Mahmoud and Abdelhameed, 2021) and other plants *Lactuca sativa* L*. Tetragonia tetragonoides*, *Portulaca oleracea* L*., Oenanthe javanica* and *Tetragonia decumbens* (Hnilickova et al., 2019; Kumar et al., 2021; Sogoni et al., 2021). In the present study exogenous Zn was applied for mitigation of NaCl stress in *Panicum miliaceum* L. and it was observed that low doses of Zn have a beneficial effect on overall plant performances (morphological and biochemical features) which is in concurrence with earlier reports wherein application of Zn mitigated salt stress in *Oryza sativa* L., *Vigna radiata* L., *Pistacia vera* L. *Ocimum basilicum* L. and *Pisum sativum* L. (Said-Al Ahl and Mahmoud, 2010; Tavallali et al., 2010; Nadeem et al., 2020; Al-Zahrani et al., 2021; Elshoky et al., 2021). Furthermore, there was a dose-dependent decrease in RWC and MSI due to salt stress and the addition of low doses of Zn significantly improved these parameters in stressed plants. In previous studies, Zn also improved RWC and MSI when applied to stress plants like *Solanum melongena* L., *Zea mays* L., *Oryza sativa* L. and *Abelmoschus esculentus* (Tufail et al., 2018; Nadeem et al., 2020; Ali et al., 2021; Raza et al., 2021; Semida et al., 2021b). An increase in growth and pigments by foliar application of Zn may be attributed to the crucial role of zinc on the biological and metabolism activity of plants. Besides, salinity stress can also negatively affect the plants by reducing the amount of photosynthetic pigments (flavinoids, total phenolics, chlorophyll, carotenoid, and anthocyanin) which has been previously observed in many plants including citrus, rice, cucumber, melon, wheat (Dionisio-Sese and Tobita, 2000; López Climent et al., 2008) (Pour et al., 2017; Hawrylak et al., 2019) (Sairam and Srivastava, 2002). The decrease in pigments contents under salt stress may be due to membrane deterioration, changes in size and number of chloroplasts, damage and injury to grana and thylakoids. The decrease of these pigments may be caused by their deterioration due to the ROS generated during salt stress (Subramanyam et al., 2019). In our study, the applications of Zn improved the photosynthetic pigments which is in accordance with several studies on wheat, tomato, rice and maize (Mathpal et al., 2015; Liu et al., 2016; Faizan et al., 2021; Rai-Kalal and Jajoo, 2021). Furthermore, it is also eveidenced in other studies that the foliar application of zinc proved positive by decreasing the injurious effect of salinity on pigments in okra plants, wheat, mungbean and rice (Tufail et al., 2018; Abou-Zeid et al., 2021; Al-Zahrani et al., 2021; Zafar et al., 2021). Similarly, Our findings revealed that applications of Zn improved the flavionod, anthocyanin, total phenolic content. Similar results were reported in many plants like *Brassica juncea*, *Hordeum vulgare and Capsicum annuum* in which zinc improved these parametrs. These pigments are essential for photosynthesis and protection of cells and the enhanced flavonoids content is directly related to better photosynthetic efficiency, superoxide radical scavenging and works as chelators in salt-stressed plants (Ahmad et al., 2017; García-López et al., 2018; Ali et al., 2022). The CSI is cardinal aspect that determines the photosynthetic ability of a plant and a higher CSI indicates greater tolerance to salt stress. In our study CSI decreased at 150–200 mM NaCl, as observed previously under various stresses in rice, mulberry and wheat (Mohan et al., 2000; Kumar et al., 2003; Babu et al., 2007; Abou-Zeid and Ismail, 2018). The application of Zn improved the CSI in accordance with previous studies on *Senna occidentalis, Solanum melongena* L.and *Triticum aestivum* L. (Farghali, 1997; Abou-Zeid and Ismail, 2018; Semida et al., 2021a). In response to salinity, the DPPH antioxidant capacity of leaf extracts, increased at 150mM NaCl and 200 mM NaCl with reference to control plants. Lower doses of Zn was found to further increased DPPH activity when given along with salt. Similar results were observed for FRAP antioxidant capacity as its activity also increased at 150mM NaCl and 200 mM NaCl with reference to control plants. Similarly, in many plants it was observed that salt increased both DPPH and FRAP activity in many plants like *Carthamus tinctorius* L., *Gossypium hirsutum* L., *Salsola baryosma*, *Trianthema triquetra*, *Zygophyllum simplex*, *Oryza sativa* L., *Nicotiana tubaccum* L.,*Crocus sativus* L. and *Triticum aestivum* L. (Xie et al., 2008; Daiponmak et al., 2010; Sharma and Ramawat, 2014; Golkar and Taghizadeh, 2018) (Mazaheri-Tirani and Dayani, 2020; Rahaiee et al., 2020; Thakur et al., 2021). For osmoprotection, plants accumulate compatible solutes such as proline under salinity stress. Proline promotes osmotic regulation by balancing cellular structures, removing free radicals and protecting cellular redox potential. In reaction to stress, proline boosting usually take places in cytosol as it adds to the osmotic adjustment. A higher accumulation of proline in plants improves their drought and salinity resistance (Surekha et al., 2014). The reducing equivalent NADPH causes reduction of glutamate to P5C, which is converted to proline. In this process NADP^+^ is generated, which is employed as an electron acceptor, inhibiting singlet oxygen and ROS formation under stress circumstances. Furthermore, NADP^+^ generated by proline biosynthesis may restore depleted NADP^+^ pools caused by Calvin cycle suppression under stress (Szabados and Savouré, 2010). In our study, PC increased under salt stress at both 150mM and 200mM NaCl. The Zn supplementation at low concentrations further increased PC content with reference to control. When Zn was administered along with salt treatments, PC improved at all concentrations which is verified by similar results obtained in *Mangifera indica* L, *Triticale* and *Triticum aestivum* L.(Arough et al., 2016; Elsheery et al., 2020; Faizan et al., 2021). Increased proline results in neutralization of the detrimental effect of stress (Hossain et al., 2010; Sofy et al., 2020) which may be due to increased activity of proline biosynthetic genes (P5CS, P5CR and OAT) and decreased activity of catabolic enzymes (ProDH and P5CDH). The P5CS enzyme, one of two main enzymes involved in proline biosynthesis from glutamate precursors, has been shown to play an important role in proline accumulation. P5CS activity in proso millet under salt stress increased at 150mM and 200mM NaCl. These findings corroborate with observations in cactus pear, carrot, rape seed, sugarcane and mustard (Han and Hwang, 2003; Silva-Ortega et al., 2008; Guerzoni et al., 2014; Kubala et al., 2015; Chandra et al., 2018). The application of low doses of Zn (1 mg/L and 2 mg/L) had a positive impact on the activity of P5CS as it increased further in comparison to control which is in accordance with various studies (Qiao et al., 2015; Luo et al., 2019; Sadati et al., 2022). Similarly, P5CR activity increased under salt stress at 150mM and 200mM NaCl as reported earlier in green gram, lentil, rice and wheat under salt stress (Misra and Gupta, 2005; Nounjan et al., 2012; Tavakoli et al., 2016), However the supplementation of Zn at lower doses further increased the P5CR activity, but the higher doses of Zn proved toxic and reduced P5CR activity. Besides, when Zn was administered with salt treatments, P5CR activity improved at lower concentrations (1 mg/L and 2 mg/L) which are in agreement with studies on exogenous application of different mitigants (Misra and Gupta, 2005; Farhadi and Ghassemi-Golezani, 2020; Zhang et al., 2020). The activity of OAT also increased under salt stress at 150mM and 200mM NaCl, besides all the doses of Zn increased OAT activity as reported previously (Da Rocha et al., 2012; Gao et al., 2019). When Zn was administered with salt treatments, OAT activity improved at lower concentrations (1 mg/L and 2 mg/L) as observed in *Arabidopsis thaliana* plantlets which showed enhanced proline content, *P5CS* mRNA and *OAT* (Roosens et al., 1998). Over expression of *Arabidopsis δOAT* gene in tobacco and rice had amplified proline content and increased stress tolerance (Roosens et al., 2002). The role of P5CDH and ProDH in catalyzing the degradation of proline is well known and in our study as expected the activities of PDH and P5CDH decreased under salt stress at 150mM and 200mM NaCl. However, Zn also helped to decreased ProDH and P5CDH activities and with combined treatment of NaCl and Zn the enzyme activities decreased further. The decreased activities leads to reduced catabolism of proline and hence accumulation of proline under stressful conditions which is in accordance with studies on chinese cabbage, rice, sweet potato and cucumber (Lopez-Carrion et al., 2008; Liu et al., 2014; Benitez et al., 2016; Naliwajski and Skłodowska, 2021).

## Conclusions

Salt stress significantly limited growth resulting in lowering of shoot length, root length, leaf area, leaf width, lead to imbalances in photosynthetic parameters, chlorophyll, membrane stability and impacted biochemical parameters related to proline biosynthesis in proso millet. Based on current research, it is evident that Zn in lower doses is very effective which provided remedial effect to salt-stressed proso millet by improving osmotic substances, antioxidant activities, photosynthetic pigments and salt stress-responsive elements. Moreover, Zn also protected proso millet through the amelioration of proline biosynthesis. The activities of enzymes governing the synthesis of proline were increased whereas the activities of the enzyme responsible for the breakdown of proline were decreased. The results proved low doses of zinc were beneficial in alleviating salt stress in proso millet and an approach like this might boost the growth and yield of plants grown under saline conditions. However, there are still many questions to be answered regarding zinc’s ability to alleviate the adverse effects of salt stress in plants. Thus deeper studies are required to answer the mechanistic role of Zn in plants and to understand the system/s governing salt stress tolerance by proline and its enzymes.

## Data availability statement

The original contributions presented in the study are included in the article/**Supplementary material**. Further inquiries can be directed to the corresponding authors.

## Author contributions

Conceptualization: NM, KA, SS, IT, AB, RR and KH. Data curation: NM, SS, RR and KH. Formal analysis: NM, KA, SS, IT, AB, RR and KH. Funding acquisition: NM, SS, AB, RR and KH. Methodology: NM, KA, SS, IT, AB, RR and KH. Software: AB, RR and KH. Validation: NM, KA, RR and KH. Visualization: RR and KH. Writing – original draft: NM, KA, SS, IT, AB, RR and KH. Writing – review & editing: KA, AB, RR, BH and KH. All authors contributed to the article and approved the submitted version.
