# Supplementary material for: Exogenous zinc mitigates salinity stress by stimulating proline metabolism in proso millet (Panicum miliaceum L.)
Source: Front Plant Sci. 2023 Mar 10;14:1053869. doi: 10.3389/fpls.2023.1053869 (PMC10036794; doi:10.3389/fpls.2023.1053869)
Supplement: Supplementary file 1 [file Table_1.docx]

**Supp. Table 1: Treatments and experimental design.**

| (T1) control | (T10) 150 mM Nacl+3mg/L Zn |
| --- | --- |
| (T2) 1 mg/L Zn | (T11) 150 mM Nacl+4mg/L Zn |
| (T3) 2 mg/L Zn | (T12) 150 mM Nacl+5mg/L Zn |
| (T4) 3 mg/L Zn | (T13) 200 mM Nacl |
| (T5) 4 mg/L Zn | (T14) 200mM Nacl+1mg/L Zn |
| (T6) 5 mg/L Zn | (T15) 200 mM Nacl+2mg/L Zn |
| (T7) 150 mM NaCl | (T16) 200 mM Nacl+3mg/L Zn |
| (T8) 150 mM Nacl+1mg/L Zn | T17) 200 mM Nacl+4mg/L Zn |
| (T9) 150 mM Nacl+2mg/L Zn | (T18) 200 mM Nacl+5mg/L Zn. |
